# Antibacterial Activity of *Corryocactus brevistylus* (Sanky) Methanolic Extract Against *Staphylococcus aureus* and *Enterococcus faecalis*

**DOI:** 10.1155/bmri/1496244

**Published:** 2026-05-12

**Authors:** Ronald Aquino-Ortega, Hugo Carrillo-Ng, Luz María Paucar-Menacho, Miguel Angel Aguilar-Luis, Wilmer Silva-Caso, Yulissa Milagros Salvatierra-Pajuelo, Ritva Repo-Carrasco-Valencia, Juana del Valle-Mendoza

**Affiliations:** ^1^ Biomedicine Laboratory, Research Center of the Faculty of Health Sciences, Peruvian University of Applied Sciences, Lima, Peru, upc.edu.pe; ^2^ Graduate School, National Agrarian University La Molina, Lima, Peru; ^3^ Academic Department of Agroindustry and Agronomy, Faculty of Engineering, National University of Santa, Chimbote, Peru; ^4^ Faculty of Food Industries, National Agrarian University La Molina, Lima, Peru

**Keywords:** antimicrobial effects, *Corryocactus brevistylus*, *Enterococcus faecalis*, sanky extract, *Staphylococcus aureus*

## Abstract

**Background:**

*Staphylococcus aureus* and *Enterococcus faecalis* are bacteria for which new antibiotics are urgently needed. *Corryocactus brevistylus* is a Peruvian cactus with an antibacterial effect that has not yet been studied.

**Objective:**

To determine the antibacterial effect of *C. brevistylus* methanol extract against *S. aureus* (ATCC 6538) and *E. faecalis* (ATCC 29212).

**Methods:**

Sanky fruits were extracted with methanol (1:2, *w*/*v*). Agar well diffusion tests were used by preparing wells with the experimental solutions cultivated in aerobic conditions for 24 h at 37°C. Six independent tests were prepared for each type of bacteria, using penicillin–streptomycin and chlorhexidine as positive controls. The MIC was determined using the microdilution method as described by the CLSI.

**Results:**

The antibacterial effect of the methanol extract was dose‐dependent, with inhibition zones of 23.33 ± 0.72 and 24.34 ± 0.55 mm against *S. aureus* and *E. faecalis*, respectively, at the highest concentration. Meanwhile, penicillin–streptomycin (10 U) and chlorhexidine produced inhibition zones comparable to the extract at the highest concentration. The MIC achieved by the extract was 15.6 mg/mL for both *S. aureus* and *E. faecalis*, while the MBC for *S. aureus* was 62.5 mg/mL and for E. *faecalis* 31.3 mg/mL.

**Conclusions:**

Weak antibacterial potency was observed in the methanolic extract of *C. brevistylus* against the strains of *S. aureus* and *E. faecalis*.

## 1. Introduction

Antibiotic resistance is currently one of the most important concerns in public health worldwide [1]. In 2014, the World Health Organization (WHO) published its first bacterial resistance global report, indicating that the treatment of common infections is becoming less effective [2]. In addition, the Infectious Diseases Society of America (IDSA) recognizes antibiotic resistance as “one of the greatest threats to human health around the world” [3]. Antibiotic resistance develops because of various mechanisms, such as lateral gene transfer, mutations in bacteria, and selective pressure due to the use of conventional antibiotics, which provides a competitive advantage for the mutated strains; likewise, the irrational use and suboptimal dosage of antibiotics contribute to this process [1]. It leads to higher mortality rates, increased treatment costs, and prolonged hospital stays [4].

In 2017, the WHO published its list of bacteria for which research on new antibiotics is urgently needed [5]. Priority Group Number 2, which consists of bacteria with a high research priority, includes antibiotic‐resistant *Staphylococcus aureus* and *Enterococcus faecium*. Within this list, both are Gram‐positive bacteria for which the development of new antibiotics is urgently required. Typically, the development of resistant bacteria occurs in the setting of healthcare‐associated infections (HAIs). Previous studies indicate that out of every 100 hospitalized patients, seven in developed countries and 10 in developing countries are at risk of acquiring a nosocomial infection [6]. A recent review showed that the prevalence of HAIs in resource‐limited countries was 15.5 per 100 patients, almost twice the prevalence in Europe [1]. HAIs are reported to cause 16 million extra days of hospital stay, 37,000 deaths, and a mean estimated expenditure of 7 billion dollars related to healthcare [7]. Both *S. aureus* and *E. faecalis* are important causes of HAIs, associated with an increase in the number of invasive procedures, such as the introduction of central venous catheters, urinary catheters, surgical site infections, and endotracheal intubation. Both bacteria are recognized causative agents of bacteremia, sepsis, skin and soft tissue infections, urinary tract infections, mechanical ventilation–associated pneumonia, and infective endocarditis [8, 9]. For example, *E. faecalis* causes approximately 97% of all infective endocarditis cases, predominantly affecting the elderly and immunosuppressed patients [3].

Currently, more attention has been given to the research on medicinal plants for the treatment of bacterial infections, due to the growing evidence of their antibacterial effect [10, 11]. The use of plants is a valuable source of bioactive compounds, due to the production of more than 100,000 secondary metabolites with multiple properties [12–14]. In Peru, around 1400 species of plants are used in traditional medicine; however, only a limited number have scientific evidence to support their use [12].

*Corryocactus brevistylus*, commonly known as “sanky,” is a native Cactaceae species grown in the Andean regions. Its chemical components have been shown to possess antioxidant properties [15]. However, its antibacterial properties have not been fully studied. Therefore, the present study is aimed at providing initial screening evidence of the antibacterial activity of the methanolic extract of *C. brevistylus* against *S. aureus* and *E. faecalis*, as a first step toward identifying potential bioactive compounds from this species.

## 2. Materials and Methods

### 2.1. Plant Material and Extracts

The fruits of *C. brevistylus* “sanky” were obtained from the “Buenos Aires” market in Nuevo Chimbote, Peru. After sanitization, they were pulped and freeze‐dried using the Labconco freeze dryer (model LYPH‐LOOK18, Germany). The freeze‐dried fruit was pulverized and immediately immersed in absolute methanol (1:2, weight/volume). The sample was incubated at room temperature and protected from sunlight for 7 days. The mixture was filtered through Whatman No. 4 filter paper, and the filtrate was evaporated at 60°C. All extracts were stored at 4°C until used. Subsequently, the extracts were dissolved in 5% DMSO. By serial twofold dilutions, a range of extract concentrations from 1000 to 3.91 mg/mL was obtained.

The taxonomic identification of the sanky fruit was performed at the San Marcos Herbarium, Museum of Natural History, Universidad Nacional Mayor de San Marcos (Lima, Peru), by morphological comparison with reference material following the Angiosperm Phylogeny Group IV (APG IV, 2016) classification system. The species was determined as *C. brevistylus* (K. Schum. ex Vaupel) Britton & Rose (Certificate Reference No. 337‐USM‐MHN‐2025).

### 2.2. Bacterial Strains

One strain of *S. aureus* ATCC 6538 and another of *E. faecalis* ATCC 29212 (Microbiologics, United States) were cultivated in brain heart infusion (BHI) agar (Oxoid, Hampshire, United Kingdom). Cultures were grown aerobically for 24 h at 37°C. Subsequently, one colony of each bacterium was resuspended in 3‐mL BHI broth and incubated aerobically for 24 h at 37°C. For the antibacterial activity assay, 20 *μ*L of the culture in BHI broth was inoculated per 100 mL of BHI agar to an approximate standard density of 0.5 McFarland, which corresponds to a concentration of approximately 1.5 × 10^8^ *C* *F* *U*/*m* *L*.

### 2.3. Antibacterial Activity of the Methanol Extract

#### 2.3.1. Determination of the Antibacterial Activity

To determine the antibacterial activity of the methanolic extract of sanky, the cup‐plate agar diffusion method was used [16]. BHI agar was autoclaved for 15 min at 121°C and cooled to 55°C. Then, it was inoculated with 20 *μ*L of the culture in BHI broth per 100 mL of BHI agar, mixed gently, and finally poured into sterile 14‐cm Petri dishes. These agar plates were incubated under sterile conditions for 3 h at room temperature. Eleven wells per plate of 8 mm in diameter and 4 mm in depth were made with a sterile borer, preserving a distance of 3 cm between them. The wells were filled with 150 *μ*L of the corresponding dilution of the methanolic extract. Penicillin–streptomycin at 10 U and 0.12% chlorhexidine (0.12*%* = 1.2 mg/mL) were used as positive controls for both bacteria [17], while 5% DMSO was used as a negative control. The Petri dishes were incubated for 24 h at 37°C. At the end of the incubation period, the inhibition zones formed were measured in millimeters using a Vernier caliper. The tests were performed in sextuplicate.

#### 2.3.2. Determination of the Minimum Inhibitory Concentration (MIC) and Minimum Bactericidal Concentration (MBC)

Serial twofold dilutions of the extract were made using a 96‐well microplate to obtain a concentration range from 1000 to 1.95 mg/mL. Bacterial inoculum of *S. aureus* and *E. faecalis* was prepared at a concentration of 1.5 × 10^8^ *C* *F* *U*/*m* *L* (0.5 on the McFarland scale), and 20 *μ*L were added per well. Each dose was analyzed in triplicate. Wells inoculated with *S. aureus* or *E. faecalis* were used as a control for bacterial growth, while tubes containing only BHI were used as a sterility control. The microplate was incubated at 37°C for 24 h, and 10 *μ*L of a 3% MTT solution was added to each well to verify cell viability. The MIC was determined as the minimum concentration at which no formazan crystal formation was observed.

After aerobic incubation for 24 h at 37°C, all samples from each well were subcultured onto BHI agar, and bacterial growth was recorded. The MBC was determined as the minimum concentration at which no bacterial growth was observed on the plate.

#### 2.3.3. Polyphenol Content Assay

Phenolic compounds were measured in the extract obtained above. The phenolic assay was based on the Folin–Ciocalteu method [18]. Briefly, 400 *μ*L of extract was mixed with 400 *μ*L of 20% Folin–Ciocalteu reagent, and 800 *μ*L of 0.3 M NaOH was added. The mixture was vortexed and incubated at room temperature for 2 min in the dark. The mixture was then centrifuged at 10,000 × *g* for 10 min in a refrigerated centrifuge at 4°C, and the contents were transferred to a 96‐well microplate. The absorbance was measured using a Synergy HTX Multi‐Modal microplate reader (Biotek, Rochester, United States) at 760 nm. A standard curve was prepared with gallic acid. The assay was performed in sextuplicate. The total phenolic content (TPC) was expressed as mg gallic acid equivalents (GAE)/g sample.

### 2.4. Statistical Analysis

Inhibition zones are reported as the *m* *e* *a* *n* ± *S* *D*. Assumptions were assessed with the Shapiro–Wilk test and Levene’s test. When appropriate, one‐way ANOVA with Dunnett’s post hoc test was applied versus the vehicle or positive control (two‐sided *α* = 0.05). Nonparametric data were analyzed using the Kruskal–Wallis test with Dunn’s correction. Dose–response was summarized with a descriptive linear regression of zone diameter versus log10[concentration] (*R* ^2^ reported). MIC determinations were performed in triplicate independent assays and summarized as modal values and ranges.

## 3. Results

### 3.1. Antibacterial Activity of the Extract

**Figure 1:**
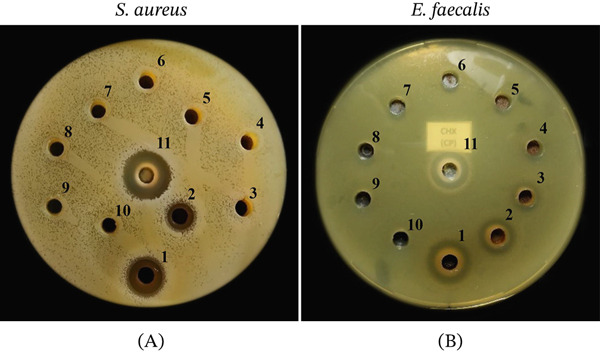
Inhibition of *S. aureus* and *E. faecalis* via *Corryocactus brevistylus* “sanky” extract versus chlorhexidine and Pen‐Strep. Inhibition halo images of sanky extract against (A) *S. aureu*s and (B) *E. faecalis*. The photographs show the distribution of the wells. Wells 1–9 correspond to the serial dilutions of sanky extract from 1000 to 3.91 mg/mL, Well 10 is 5% DMSO, and Well 11 is chlorhexidine. Pen‐Strep inhibition zones are not shown.

The antimicrobial effect of *C. brevistylus* extract was tested by the cup‐plate agar diffusion method using *S. aureus* and *E. faecalis*. In both strains, working concentrations were in the range of 1000 to 3.91 mg/mL (using twofold serial dilutions). In both cases, the antimicrobial dose–response of the sanky extract against the Gram‐positive *S. aureus* and *E. faecalis* was observed (Figure 1A,B). However, the inhibitory effect can be seen graphically at concentrations of 250 and 125 mg/mL, respectively. At lower concentrations, the effect was minimal or zero.

**Figure 2:**
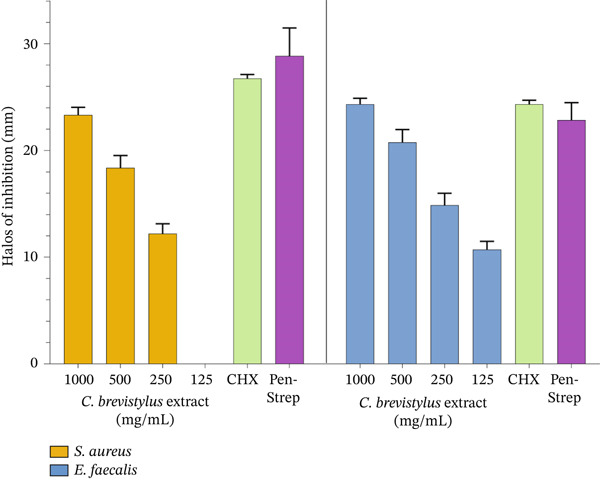
Bar graphs of the inhibition halos measured in the antimicrobial susceptibility test.

To quantify the inhibition halos of *S. aureus* and *E. faecalis* bacteria, Figure 2 shows bar graphs of the inhibition halos measured in the antimicrobial susceptibility test. In this figure, the halo of inhibition is larger in the case of *E. faecalis* compared to *S. aureus* at all dilutions tested (from 1000 to 125 mg/mL).

**Figure 3:**
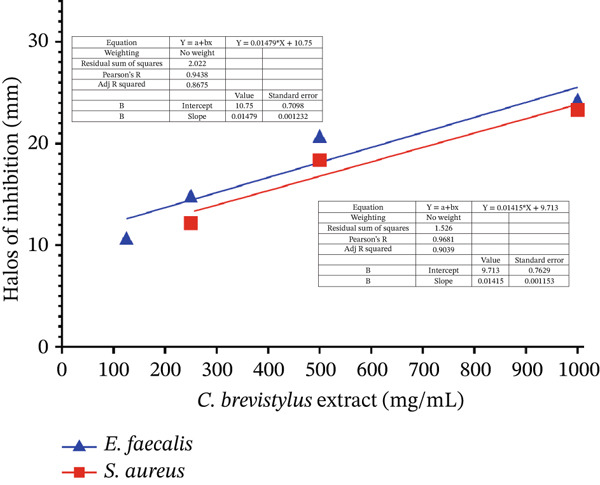
Linear fit of the inhibition halos produced by the *C. brevistylus* extract.

The measured values of the halo of inhibition associated with the bacteria *S. aureus* and *E. faecalis* can be represented by a linear fit with correlation factors *R* ^2^ of 0.97 and 0.94, respectively (Figure 3). This fact suggests that there is a direct correlation between the concentration of the extract and the antimicrobial activity through the formation of inhibition halos. Table 1 summarizes and compares the results of the antimicrobial assay obtained. Furthermore, it shows the inhibition zones obtained by chlorhexidine and penicillin–streptomycin.

**Table 1:** Comparison of inhibition halos at different concentrations of *Corryocactus brevistylus* “sanky” methanolic extract.

Microbial strain	CHX1.2 mg/mL	Pen‐Strep10 U	Various concentrations of the extract (mg/mL)									ANOVA*p*value∗
			1000	500	250	125	62.5	31.3	15.6	7.8	3.91	
*S. aureus*	26.8 ± 0.4^a^	28.5 ± 2.6^a^	23.3 ± 0.1^b^	18.4 ± 1.2^c^	12.2 ± 0.9^d^	0.0 ± 0.0^e^	0.0 ± 0.0	0.0 ± 0.0	0.0 ± 0.0	0.0 ± 0.0	0.0 ± 0.0	< 0.001
*E. faecalis*	24.3 ± 0.4^a^	22.9 ± 1.2^a^	24.3 ± 0.6^a^	20.8 ± 1.2^b^	14.9 ± 1.2^c^	10.7 ± 0.8^d^	0.0 ± 0.0	0.0 ± 0.0	0.0 ± 0.0	0.0 ± 0.0	0.0 ± 0.0	< 0.001

∗Different superscript letters (a, b, c, d, and e) indicate statistically significant results of the one‐way ANOVA and Dunnett’s test (*p* < 0.05).

The MIC of the methanolic extract of sanky was determined in the microdilution test for *S. aureus* and *E. faecalis* strains, while the MBC was determined by subculture based on the MIC (Table 2).

**Table 2:** Minimum inhibitory concentration (MIC) and minimum bactericidal concentration (MBC) of the *Corryocactus brevistylus* methanolic extract against *Staphylococcus aureus* and *Enterococcus faecalis.*

Response∗	Various concentrations of the extract (mg/mL)								
	500.0	250.0	125.0	62.5	31.3	15.6	7.8	3.9	1.9
MIC *S. aureus*	*−*	*−*	*−*	*−*	*−*	*−*(MIC)	+	+	+
MBC *S. aureus*	*−*	*−*	*−*	*−*(MBC)	+	+	+	+	+
MIC *E. faecalis*	*−*	*−*	*−*	*−*	*−*	*−*(MIC)	+	+	+
MBC *E. faecalis*	*−*	*−*	*−*	*−*	*−*(MBC)	+	+	+	+

∗Response means the indicator of the tests performed (formation of formazan crystals for MIC determination and bacterial growth for MBC determination). (−) No growth. (+) Growth.

The MIC achieved by the extract was 15.6 mg/mL for both *S. aureus* and *E. faecalis*, while the MBC for *S. aureus* was 62.5 mg/mL and for *E. faecalis* 31.3 mg/mL.

The TPC assay was performed using the Folin–Ciocalteu colorimetric method. The results showed that the sanky extract contained a TPC of 27.20 ± 2.88 mg GAE/g extract.

## 4. Discussion

According to the WHO, approximately 15% of hospitalized patients are at risk of acquiring HAIs [6]. *S. aureus* and *Enterococcus* spp. are among the most prevalent Gram‐positive bacteria causing HAIs at 15.9% and 9.6%, respectively. Moreover, it has been reported that both bacteria showed an alarmingly high rate of antibiotic resistance: 41.2% of the *S. aureus* isolates were methicillin resistant, and 10.2% of the *Enterococcus* spp. isolates were vancomycin resistant [17, 19], so the treatment against both bacteria is becoming less effective [20, 21].

The antibacterial activity observed in the present study should be interpreted cautiously. Although the crude extract inhibited the growth of *S. aureus* and *E. faecalis*, the MIC values remained in the mg/mL range, which is generally considered weak activity for crude plant extracts and is far below the potency expected for conventional antibiotics acting in the *μ*g/mL range. Likewise, the MBC values indicate a limited bactericidal effect. Therefore, the extract in its crude form is unlikely to represent a realistic systemic antibacterial candidate. Instead, these findings mainly suggest the presence of one or more bioactive constituents, possibly at low abundance, that deserve further isolation and characterization.

Interest in plant‐derived antimicrobials has increased in response to the global rise of antibiotic‐resistant bacteria and the long‐standing use of medicinal plants in traditional medicine [13, 22]. Their potential lies in their chemical diversity and in the multiple mechanisms through which they may affect microbial growth and susceptibility. However, antibacterial efficacy, safety, and the risk of resistance development cannot be generalized and should be assessed individually for each extract or purified compound.

In this context, the study of *C. brevistylus* is relevant because no previous reports were found describing the antibacterial activity of its extracts. Although the activity observed here was modest, these results provide an initial basis for future bioassay‐guided fractionation and the identification of the compounds responsible for the detected effect.

The determination of inhibition halos and MIC results obtained in this study showed that *E. faecalis* was more sensitive than *S. aureus*. *E. faecalis* presented inhibition halos ranging from 24 to 10.7 mm, whereas *S. aureus* showed halos between 23.3 and 12.2 mm, respectively (see Table 1). Sánchez et al. [23] evaluated methanolic extracts of *Opuntia ficus* against *E. faecalis* and *S. aureus* and obtained inhibition halos of smaller diameter than those found in this study (15 and 16 mm, respectively). However, comparisons between studies should be interpreted cautiously because the agar well diffusion assay is considered a qualitative technique and has a series of technical limitations, such as diffusion limitations or agar composition. Despite these limitations, inhibition halos greater than or equal to 10 mm in diameter are commonly used as a criterion to consider extracts as active [24]. In MIC assays, both *S. aureus* and *E. faecalis* showed the same inhibitory concentration, whereas the MBC values differed. *S. aureus* required twice the concentration of extract compared with *E. faecalis* to achieve bactericidal activity, suggesting that *S. aureus* requires a higher concentration of sanky extract for complete eradication. This could be possible because *S. aureus* has more developed tolerance mechanisms, such as the generation of subpopulations called “persisters” [25]. Furthermore, the presence of efflux pumps such as NorA and its antioxidant systems could confer greater resistance to plant‐derived bioactive compounds [26]. In contrast, the susceptibility of *E. faecalis* could be associated with its metabolic limitations in dealing with external agents. These findings highlight that MIC values alone may not accurately predict the bactericidal potency of a plant extract and that the combined interpretation of MIC and MBC provides a more comprehensive understanding of antimicrobial activity.

Broadly, our results agree with a wide variety of studies published regarding the antibacterial effect of plant extracts derived from other Cactaceae species against Gram‐positive bacteria. Souza et al. [27] and Garcia et al. [28] evaluated the antimicrobial activity of *Pereskia aculeata* and determined that the chloroform and ethanolic extracts exhibited activity against Gram‐positive bacteria, highlighting their effect against *S. aureus*. Similarly, Benramdane et al. [29] revealed that the lipophilic extract of *O. ficus*‐*indica* was effective against *S. aureus*, with inhibition halos that reached 23 mm, which is comparable to the halo diameters observed in this study. These findings suggest that extracts from Cactaceae species may exhibit measurable antibacterial activity, possibly related to their diverse phytochemical composition.

The MIC values obtained for the crude extract of *C. brevistylus* are relatively high according to commonly used phytochemical criteria for plant extracts. In the evaluation of crude extracts, activity is assessed using specific thresholds (e.g., *M* *I* *C* < 100–625 *μ* *g*/*m* *L* as significant/moderate), not against potencies of purified antibiotics (*μ*g/mL). Our values are classified as weak activity, rendering the extract unviable as a systemic antibacterial agent. Instead, these results should be interpreted as preliminary screening evidence suggesting the presence of bioactive compounds that may occur in low abundance within the extract. Consequently, further studies should focus on bioassay‐guided fractionation to enrich active fractions, the identification of the compounds responsible for the activity, the evaluation of possible synergistic effects with conventional antibiotics, and the exploration of mechanisms of action. In addition, optimization of extraction procedures using alternative solvents or green extraction technologies (e.g., ultrasound‐ or microwave‐assisted extraction) could improve the recovery of bioactive compounds and potentially enhance the observed antibacterial activity.

This study did not investigate the exact mechanism by which sanky exerts its antibacterial effect. However, the antibacterial activity observed may be associated with the presence of polyphenolic compounds, as these molecules have been reported to contribute to enzyme inactivation, act as prooxidants, inhibit protein synthesis, and disrupt cell membranes. Areche et al. [15] identified 38 compounds in the ethanolic extract of sanky with potential antioxidant and gastroprotective properties. Among these, flavonoids such as rutin, quercetin, and isorhamnetin were detected, and their antimicrobial activity has been reported in previous studies [30]. Similarly, other members of the Cactaceae family, such as *Opuntia* spp., contain relatively high levels of rutin and isorhamnetin and have been reported to exhibit greater activity against Gram‐positive bacteria than Gram‐negative bacteria [6].

However, it should be noted that the present study did not determine the total flavonoid content nor perform detailed chemical profiling of the methanolic extract evaluated. Therefore, any mechanistic interpretation remains preliminary and should be considered a hypothesis supported by the TPC and previous phytochemical reports.

This study evaluated the TPC of the sanky extract, obtaining a value of 27.20 ± 2.88 mg GAE/g, which exceeds the 24.34 ± 3.67 mg GAE/g previously reported by Areche et al. [15]. This variation could be attributed to different factors such as the geographical origin of the sanky, crop conditions, or the extraction method used. When comparing our findings with those of other cacti, the TPC of sanky extract was found to be considerably higher than that of *O. ficus-indica* pears [31], which present values between 9.64 and 12.28 mg GAE/g. On the other hand, the TPC value of sanky extract is lower than that of Peruvian cacao peel (*Theobroma cacao*), which can reach values of 51.52 mg GAE/g [32]. Despite these differences, these findings indicate that the sanky extract contains a considerable amount of phenolic compounds and may represent an interesting source of phytochemicals for further investigation.

The inhibition zone diameters and MIC/MBC results indicate that sanky extract exhibits measurable antibacterial activity in vitro, although the relatively high MIC values suggest limited antibacterial potency for a crude extract. These findings may therefore reflect the presence of bioactive compounds occurring at low abundance within the phytochemical matrix.

Finally, to our knowledge, this is the first study to evaluate this plant extract against commercial strains of *S. aureus* and *E. faecalis*, despite the limited previous information available regarding the antibacterial effect and clinical relevance of *C. brevistylus*. Further research is needed to assess its activity against clinical and resistant strains and to identify the specific bioactive compounds responsible for the observed antibacterial effect, as well as their safety and potential toxicity.

## 5. Conclusions

The methanolic extract of *C. brevistylus* (sanky) showed measurable antibacterial activity against *S. aureus* and *E. faecalis* under in vitro conditions. However, the relatively high MIC and MBC values indicate weak antibacterial potency for a crude extract and do not support its consideration as a realistic systemic antibacterial drug candidate in its current form. Instead, these findings should be interpreted as exploratory evidence suggesting that the extract may contain one or more bioactive compounds present at low abundance within a complex phytochemical matrix.

Despite this limitation, the study provides an initial scientific basis for the antibacterial evaluation of *C. brevistylus*, a species for which such evidence remains scarce. Future research should focus on bioassay‐guided fractionation to isolate and identify the compounds responsible for the observed activity, as well as on studies addressing their mechanisms of action, possible synergistic interactions with conventional antibiotics, and safety profile. These approaches will be essential to determine whether this plant may represent a relevant source of antibacterial adjuvants or novel phytochemical leads rather than a direct therapeutic agent.

## Funding

This study was funded by Dirección de Investigación‐Universidad Peruana de Ciencias Aplicadas (UPC‐A‐070‐2020) and Concytec‐World Bank (02‐2018‐FONDECYT‐BM).Ronald Aquino‐Ortega acknowledges the financial support of the Concytec‐World Bank Project, through its implementing unit, the National Fund for the Development of Science, Technology and Technological Innovation (Fondecyt) (Funding Agreement No. 02‐2018‐FONDECYT‐BM), for his research work.

## Disclosure

We declare that a preliminary version of this manuscript was presented in abstract format at the annual meeting of the American Society of Tropical Medicine and Hygiene in 2024 and was published in its book of abstracts by IMED 2021 under the consideration of the Committee on Publication Ethics (COPE).

## Ethics Statement

The authors have nothing to report.

## Consent

The authors have nothing to report.

## Conflicts of Interest

The authors declare no conflicts of interest.

## Data Availability

Data are available on request from the authors.
